# Adolescents’ Perceptions and Attitudes towards Traditional and Electronic Cigarettes—Results of Focus Group Interviews

**DOI:** 10.3390/ijerph20021438

**Published:** 2023-01-12

**Authors:** Agnieszka Wojtecka, Olga Kalinowska-Beszczyńska, Anna Tyrańska-Fobke, Dorota Kaleta, Małgorzata Wojnarowska, Marlena Robakowska, Łukasz Balwicki

**Affiliations:** 1 Department of Public Health and Social Medicine, Medical University of Gdansk, 80-210 Gdansk, Poland; 2 Department of Hygiene and Epidemiology, Medical University of Lodz, 90-647 Łódź, Poland; 3 Center for Competence Development, Integrated Care and e-Health, Medical University of Gdansk, 80-210 Gdansk, Poland

**Keywords:** cigarettes, young adults, perception

## Abstract

There is general agreement among scholars and policymakers that efforts to reduce smoking and prevent nicotine use should be intensified and focused on the most vulnerable part of society—adolescents. Regardless of common knowledge about the health consequences of smoking, according to data from 2020, about 51% of children in Poland had their first contact with smoking at the age of 15 and older. The objective of this research is to investigate motivations to start smoking or vaping, patterns of using tobacco products, perceptions of smoking/vaping and smokers/vapers, as well as attitudes toward nicotine addiction. The broad aim is to reflect on youth perceptions of nicotine use. A qualitative approach has been applied, utilising focus-group interviews. The sample included six focus groups, consisting of smokers and non-smokers of both genders, situated in three different cities in Poland. The interviewees were adolescents ages 16–18, attending high schools, technical schools, or vocational education. Interviews were transcribed and further analysed using the thematic analysis approach. This research enhances previously reported results by revealing new insights into the strategies applied by adolescents to mitigate financial barriers arising from high costs of nicotine products, as well as highlighting methods used to avoid the traditional antismoking messages placed on packaging.

## 1. Introduction

Despite continuous efforts to reduce smoking and prevent new nicotine initiations, research shows that children and adolescents in their early years of development still experiment with smoking. Even 10-year-olds gain their first experience in smoking and some become regular smokers around the age of 15 [1,2]. Reitsma et al. conducted an extensive study on the prevalence of smoking-tobacco use and initiation among young people, analysing over 3000 tobacco surveys conducted between 1990–2019 from 204 countries and territories around the world and found two important general patterns [2]. First, the majority of tobacco smokers (65.5%) start smoking at the age of 15–20, and this pattern of smoking initiation persists in most countries. Second, over the last 30 years, the prevalence of smoking among young people globally has decreased by about 33% and in Latin American and Caribbean countries by as much as 50%. On the other hand, there was a slight increase in smoking among women in the regions of North Africa and the Middle East and Southeast Asia and Oceania. It should be noted that while there is a global decline in smoking, it is too slow to end the epidemic in a single generation [2].

Poland does not differ from other countries in terms of early smoking initiation. Despite common knowledge about the health consequences of smoking, according to data from 2020, about 51% of children in Poland had their first contact with smoking at the age of 15 and older, and 8% started smoking before the age of 10 [3]. In the 15–18 age group, approximately 23% of Polish teenagers reported having smoked at least 40 times in their lives, while over 44% of teenagers in this age group said they had never smoked before [3]. Poland ranks in the top ten countries with the highest prevalence of smoking among young people aged 13–15, with about 22% of teens in this group smoking cigarettes [3].

A comprehensive literature review by Ren and Loflipour, including data from publications between 1968 and 2018, shows the physiological effects of nicotine use among adolescents [4]. The results of the review show that nicotine uniquely activates the adolescent brain’s reward system, as children and teens have an overactive response to reward, which promotes addiction. The review also showed that nicotine exposure before the age of 17 was associated with an increased risk of alcohol use compared to the same exposure after the age of 17. In addition, nicotine use by adolescents increases the likelihood of using other psychostimulants. People who started smoking before the age of 15 were 80 times more likely to use cannabis and illicit drugs.

The most commonly observed risk factors for smoking at a young age include having family members [5,6] or friends who are smokers [6,7,8,9,10]. Other risk factors include stress, depression, and vulnerability to peer influence [9]. In turn, protective factors against smoking include good academic performance, being a member of a large family, physical activity and involvement in sports, high self-esteem, personal skills such as the ability to stand against pressure, and having other health problems [9,11,12,13].

Another problem with taking nicotine is the use of e-cigarettes. The main predictor of e-cigarette use by the Polish youth is previous smoking of traditional cigarettes. Other risk factors include the belief that it is less harmful and having parents who smoke [14].

Actions aimed at protecting children and young people from nicotine initiation can be a big challenge. Planning such activities carries many risks, and improperly designed interventions can have effects that are difficult to predict by adult observers. Pupils can respond to a school smoking ban by shifting the smoking habit to another place and time [15]. In the case of a ban on the sale of cigarettes, young people are ready to use various tactics of forming alliances with older colleagues to ensure easy access [16]. The reaction to the increase in cigarette prices may be attempts to increase one’s income instead of the expected reduction in consumption [17]. Therefore, in order to avoid failed or counterproductive interventions, it is important to know the experiences, lifestyles, and views of young people.

The starting point for planning prevention programs should be learning about the basic characteristics of the group for which they will be intended and identifying the needs of the target population. The content and forms of activities should be appropriately matched to the characteristics of the recipients of the intended intervention. In the case of adolescents, understanding their situation by getting to know their experiences, interests, idols, lifestyle, style of relationship-building, ways of communicating, and home and economic situation is extremely important for the correct identification of high-risk groups. Subsequently, this knowledge should be used to guide broad-based actions and targeted interventions resulting in the reduction, and preferably cessation, of tobacco use in adolescence and adulthood [9]. It should also be noted that programs that involve peers and that last throughout adolescence produce the best results [18,19,20].

Based on these previous studies and on the results of behavioral and biological studies, it has been observed that adolescents are particularly susceptible to addiction and most adult smokers regret that they started smoking [21,22,23]. This paper aims to broaden the current knowledge of Polish adolescents’ attitudes and perceptions of tobacco use and cigarette smoking.

At the theoretical level, by enhancing the general understanding of the context of youth smoking. Our research investigates motivations to start smoking or vaping, patterns of using tobacco products, perception of smoking/vaping and smokers/vapers, as well as attitudes toward nicotine addiction, and thus, aims at reflecting the youth’s understanding of the phenomena of nicotine use.

At the practical level we believe that the results obtained could provide further guidance for formulating effective actions that prevent smoking and vaping among young people, as well help addicts to quit nicotine use.

## 2. Materials and Methods

The qualitative approach has been selected as the most suitable method to achieve the aim of the research. As the focus of the investigation is to describe and understand the perspective of adolescents, the interpretative feature of qualitative research is vital. Further, the fact that it is case-oriented provides an opportunity to display the data in a real-life context as well as, to take that context into account [20], comprehensively embracing the researched phenomenon. Moreover, use of an inductive approach allows for the ability to generate theory that can provide a solid background for future qualitative research that could test the proposed theory.

### 2.1. Research Design and Sample

The study was a part of the national program for combating the health consequences of using tobacco and related products, financed by the National Health Program 2016–2020 from the Fund for Solving Gambling Problems at the disposal of the Minister of Health in Poland. The qualitative part discussed was devoted to exploring and understanding adolescents’ attitudes toward smoking and nicotine products. The age category of ‘adolescents’ follows the WHO definition and ranges between 10 and 19 years old. Yet for research purposes, the age frame is limited to the range that reflects attendance in secondary education. The focus group interviews (FGI) were selected as the tool to collect data.

A set of subjects were formulated that would allow researchers to fulfil the aim of the project, and leading questions were designed. Examples of questions can be found in Table 1 below. However, it should be noted that those questions were used more as a trigger for discussion, thus participants were expected to elaborate from them in more detail.

The sample consisted of adolescents aged 16–18 years old, attending either high school, technical school or vocational training. Focus group participants were divided by gender (male/female) and by use of nicotine products (nicotine users/non-nicotine users). Those classified as ‘nicotine-users’ used a variety of products, such as cigarettes, e-cigarettes, or heated tobacco products, on a daily basis for at least the last six months; whereas, those classified as ‘non-nicotine users’ have never used any products containing nicotine. The participants classified as ‘nicotine users’ experienced a variety of nicotine containing products, either in different phases or concurrently.

The sample had not been further divided into subcategories of ‘vapers’, ‘smokers’ or ‘dual users’. All participants were classified together as ‘nicotine users’.

The details of the research design and the formal requirements had been revised and approved by The National Institute of Public Health PZH-National Research Institute Bioethical Committee Board.

### 2.2. Data Collection

The participants were recruited and interviewed by a professional company specialising in market research. The interviews were conducted in designated facilities by professionals having experience working with teenagers and young adults.

The data were collected via focus group interviews conducted during the period between 29 and 30 October 2022. A total of six focus group sessions were conducted: two sessions with female nicotine users; two sessions with male non-nicotine users; one session with male nicotine users; and one session with female non-nicotine users. The focus group interviews distribution you can find in Table 2. The sessions took place in three cities: Lublin, Kielce, and Warsaw. Each session took up to 120 min and included eight participants in each group (total number of participants 48).

A focus group interview guide was created with a series of open-ended questions, examples of which are given in Table 1. A trained moderator was present at all times to oversee and facilitate the process. The focus group participants were informed about the research goals and told that they are going to be observed and that their responses would be recorded. Informed consent was obtained from all participants.

### 2.3. Data Analysis

All of the focus group interviews were recorded, transcribed into Polish, and the data entered into the NVivo qualitative data analysis software.

The collected data were then coded by two independent researchers and analysed using the thematical analysis approach. The thematical analysis allowed for systematic identification, organization, and insight into patterns of themes in the qualitative dataset [24]. More importantly it allowed us to make sense of collective or sharing meanings and experiences, which was crucial to achieving the objective of the research [24]. Features such as theoretical independence and flexibility in approaching the data paired with suitability for a variety of data types [25] and added to the overall utility of the applied method.

## 3. Results

The obtained results can be grouped under four sub-themes: (1) the differences in perception of traditional cigarettes compared to e-cigarettes; (2) motivation to start and continue using nicotine; (3) patterns of using nicotine products; and (4) insights into young adults’ attitudes toward anti nicotine campaigns. Details and data from the focus group interviews about each of the elicited sub-themes is presented below.

### 3.1. Traditional Cigarettes Versus E-Cigarettes

Generally, e-cigarettes are perceived by young adults as a ‘less serious’ version of traditional cigarettes containing tobacco. They often became a first choice while starting using nicotine products as well as the choice of the younger smokers.

‘*…the younger ones, every second one has an electric.*’

(NNU Female R3K)

Moreover, e-cigarettes can be treated as gadgets and, therefore, can be regarded as a part of fashion and style. In some social circles, e-cigarettes can be a source of competition. Some respondents indicated that there are a number of social media clips promoting tricks that can be performed with the exhaled smoke which their peers try to mimic.

‘*…there are different types of them (e-cigarette gadgets), some look cool, some less cool.*’

(NNU Female R1K)

‘*He would sit like this for the whole day and make those circles, or he sees something on the internet and wants to do the same with the smoke.*’

(NU Female R5L)

On the other hand, there is no doubt among young users that the traditional cigarettes are easier to handle as they are less demanding in terms of ‘service’ or additional devices that are required for using e-cigarettes.

*‘… The electric one may break, the switch might be dirty or the heater may be broken.*’

(NU Female R8W)

There is a widely shared agreement among both smokers and non-smokers that the e-cigarettes are less disruptive compared to the traditional ones (containing tobacco). In part, this is because they smell less. We may also observe a tendency to believe the e-cigarettes to be the ‘healthier’ alternative if one already smokes.

*‘…so when you are with your friends and they smoke the traditional ones, you have a healthier alternative.’*

(NU Male R2L)

### 3.2. Motivation to Start and Continue Using Nicotine Products

When asked to recall their first experiences with smoking and using nicotine, most participants indicated that they had their first cigarette before the age of 14. They did not have much influence on the type of the cigarette, whether it was a traditional or e-cigarette, as often it was offered to them by a third party—usually a peer or older sibling. As to the triggers for starting to smoke while young, adults cited factors such as curiosity and peer pressure. There was no explicitly indicated fear of being rejected by the group, yet the opinion of the peer group played a critical role.

The first impressions of smoking/vaping were reported as generally unpleasant. Dizziness, coughing, and bad taste were indicated as the most memorable side-effects. Nevertheless, they decided to keep trying.

A number of reasons to continue smoking or vaping were pointed out by the participants. A great majority indicated the socializing aspect. Smoking/vaping is perceived as a way to join a new group or maintain relationships with an established one. However, the social aspect refers exclusively to the smoking/vaping peers. It seems that the smokers/vapers and non-smokers/non–vapers do not mingle. This is particularly distinct in reference to traditional tobacco cigarettes. Interestingly, the non-smokers declare no reluctance toward their smoking peers, yet they tend to stay apart.

*‘… Smoking is the reason to go out and have a chat.*’

(NNU Male R4K)

‘*… Those who don’t smoke stay in school, smokers go out.*’

(NNU Male R2K)

*‘… Maybe some people are ok with it, but it puts me off (…) a girl should smell nice.*’

(NNU Male R2K)

Among other reasons to smoke, stress and other emotional factors were two of the most frequently indicated.

*‘… Always when I’m angry (…) my hand is in the pocket and the cigarette is out.*’

(NU Male R5L)

‘*… When we’re stressed, we can smoke three cigarettes in a row (…).*’

(NU Female R1W)

When asked about the source of stress, participants indicated school, family, friends, and personal failures. On the other hand, smoking is associated with one’s circumstances, such as engagement in social life or partying, and often accompanied by consumption of alcohol. Besides providing pleasure, smoking/vaping as an activity by itself is also perceived as a way to kill time.

### 3.3. Patterns of Use

When considering patterns among young adults, especially in reference to e-cigarettes, it is clear that they are used almost everywhere and most of the time. Among the most frequently cited places were the school and the school’s neighborhood, the street, home, clubs, and even a gym.

Nevertheless, there were certain circumstances in which the respondents declared to refrain from smoking/vaping. The motivations could have a source in external factors, such as the presence of parents or a strict prohibition that could not be bypassed easily, or they were internally motivated, for example, while being sick or feeling that smoking is inappropriate in a given situation.

As the cost of cigarettes is an important component in young people’s budgets, it is sometimes indicated as a potential reason either to limit or quit smoking.

‘*I think, that the price is going up, and that money, this 16 zloty, (equivalent of 3.5 USD) I spend every day for a package. I could save for a different expenditure (…).*’

(NU Female R8W)

Although the high price of cigarettes may act as a barrier to smoking, the respondents had strategies to overcome it. Sharing costs of purchase—for example, in case of liquid tobacco—was discussed as one of the applied tactics.

Few of the respondents reported attempts to quit smoking. For those respondents who did report quitting attempts, in all cases they sooner or later returned to their old habits.

‘*I managed a week without a cigarette. Then someone annoyed me, so I went and bought a pack* (of cigarettes).’

(NU Male R5L)

‘*When I went to the institution (The type of institution was not indicated), I didn’t feel like smoking. There was no money for cigarettes. I didn’t smoke. I’ve jumped out of the institution and I’m smoking again.*’

(NU Male R3W)

In the case of female respondents, besides serious illnesses, they indicated pregnancy as a potential motivation to quit smoking.

‘*(…) so without pregnancy there is no reason to quit. Unless there is a serious illness related to lungs or thought.*’

(NU Female R3W)

Overall, the majority of respondents did not take quitting smoking as a serious or even proximate goal. Moreover, at this stage, many respondents indicated that they did not intend to stop smoking in adulthood.

### 3.4. Perception of Anti-Nicotine Campaigns among Young Adults

Considering the general knowledge of young adults, both smoking and non-smoking youths are aware of the health hazards related to the use of cigarettes. However, the non-nicotine users seemed to be more inclined to understand these health risks. We observed a common belief among youth that e-cigarettes are less harmful compared to the traditional ones. This assumption arises from the fact that the packaging is neutral compared to traditional cigarettes. Moreover, sometimes the liquids are advertised as nicotine free, thus creating a false impression of being harmless.

‘*…I have started with electric cigarettes earlier, because there was a type without nicotine.*’

(NU Female R2W)

While assessing the effectiveness of including drastic photos on the packaging of traditional cigarettes, the results show that the photos did not have the intended effect of decreasing of nicotine consumption. A number of respondents indicated that those photos are neither shocking nor convincing for them. This is mainly because they do not know anyone who had any of the depicted diseases and are thus unable to personally connect with such potential consequences. As the effects presented are primarily the result of long-term smoking, the proximity of the effect is too distant, so it becomes too abstract to be relevant to young adults.

‘*… But those (pictures) are the extreme cases. (…) everything may lead to an illness, but you don’t really hear about such cases.*’

(NU Female R2W)

In some cases, smokers who were more sensitive and felt disgusted by the pictures either bought only a particular type of “picture” or used special covers to hide the photo printed on the packaging. An adverse reaction was also observed—one of the respondents mentioned a friend who was actually collecting those pictures and treating it as a hobby.

‘*… those (pictures) are disgusting, (…) I have a special cover, a black one with a nice picture.*’

(NU Female R4W)

## 4. Discussion

This research led to new results, which show how interventions aimed at reducing the availability of cigarettes are circumvented by young people. The high cost of cigarettes is intended to discourage their purchase. Yet, it turns out that this barrier is overcome by sharing the cost of the package among the members of the group of young smokers. This solution allows for the continuous distribution of small portions of cigarettes. As a result, the intervention’s objectives are only partially attained: the consumption is reduced, yet not fully stopped. Another interesting result is the fact that young people ignore the message conveyed by the pictures on cigarette packs. The purpose of the pictures is to educate consumers about the harmful effects of smoking and to discourage smoking through photos of people affected by tobacco-related diseases. It turns out that young people do not relate the potential harmful consequences of smoking to themselves and do not perceive these consequences as real. In addition, e-cigarette packs are free of these types of messages, which further undermines the power of educational efforts regarding the harmful effects of smoking.

Other results of the study show that, in general, young Poles share key characteristics and attitudes related to smoking with their peers from other countries.

We identified in Poland a set of factors that influence the onset and continuation of smoking that could be classified similarly to the research conducted by Peterson, Harell et al. [26]. Similar to the Uruguayan survey, we identified intrapersonal and interpersonal factors, such as peer modelling behavior—miming the group, as well as the importance of intrapersonal factors—such as ‘being seen’ as a member of a social group. In adolescence, there is a change of authority. The rules imposed by adults are challenged by young people. Rules of conduct, including affiliation or rejection, are determined by the youth’s group leaders. As such, cigarettes may be one of the factors uniting the group. Therefore, smoking makes it easier to form and maintain relationships. Thus, peer pressure is an interpersonal risk factor for smoking. We also observed that, since group acceptance is an important factor, young adults have difficulties setting boundaries and rejecting smoking. This is consistent with findings reported by Tyas [9] on the limited interpersonal skills of young adults.

In addition to the problem of smoking initiation, there is also the question of continuing to smoke. This leads to the problem of addiction at a young age. According to the American Cancer Society, the sooner you start smoking, the more likely you are to become physically and psychologically addicted to nicotine [27]. People who smoke regularly for several weeks have withdrawal symptoms when they try to stop, which is why it is difficult for teenagers to stop smoking. Our research empirically supports this statement, as the respondents reported difficulties with quitting smoking, as well as returning to the smoking habit after a while.

The problem is compounded by the nicotine addiction. E-cigarette manufacturers encourage the young to consume cigarettes with candy and fruit flavors [28]. Although several companies claimed that a few of their products do not contain nicotine, some nicotine was found in many of their products [27]. It has been observed that people who start using e-cigarettes in the long-term switch to traditional cigarettes. This creates particularly high risks for young adults, as e-cigarettes are perceived as non-serious gadgets and, therefore, harmless. However, it turns out that the use of e-cigarettes has negative health effects, including damage to the lungs [28]. More recent research also points to cardiovascular, immune, and neurodevelopmental effects. Many of these effects are likely dose-dependent [29]. Therefore, urgent action is needed to reduce or completely limit the initiation of e-cigarette use and to establish smoking-cessation support.

The research presented has several limitations. Mainly, it was based in cities; therefore, these trends may not extend to the perceptions and attitudes of young people living in rural areas, which might differ due to stronger influences of the community where they live. Furthermore, the distribution of group characteristics was not equal, with more of a focus on female nicotine users than male nicotine users. In order to overcome some of the bias related to the need to conform to group standards, the FGI were conducted by a professional moderator in a specially arranged environment that created a safe place for sharing thoughts and feelings. Moreover, all participants were treated as a homogenous group of ‘nicotine users’. Therefore, individual characteristics of ‘vapers’, ‘smokers’ and ‘dual users’ were not identified. Further research is required to clarify the above issue.

## 5. Conclusions

The collected data indicate several key issues. First, in order to communicate effectively with the young adult population, we need to start to listen more carefully to their needs and better understand their perspectives and attitudes around tobacco and smoking. This is crucially important as the research indicates that the popularity and perception of e-cigarettes have serious future consequences [30]. Jenssen & Walley highlight that using e-cigarettes increases the likelihood of switching to traditional cigarettes. Additionally, we observed that e-cigarettes are perceived as a fun product dedicated to younger users, thus, increasing the risk of evolving into long term addiction in the larger population.

Adults and the authorities responsible for creating the antismoking campaigns need to take into account the young adults’ perspective as the principal factor to be addressed. A vivid example can be drawn from the declared motivations to smoke. We should address the key problems to which smoking is an answer—such as ability to cope with stress or to create relationships with new and established peer groups. We also need to reconsider and revise the current channels of communication as well as the content of messages. The existing model is clearly inefficient and does not relate to aspects important to young adults today.

A potential further course of action is to understand why the measures to discourage smoking are failing, largely ignored, or are not perceived as a real threat. Perhaps consideration should be given to qualitative research on reinforcing incentives to quit using any nicotine-containing products because young people are susceptible to advertisements of new products and are eager to experiment with new ones. It is especially necessary to thoroughly recognize the needs of young people and their susceptibility in order to prevent the initiation of nicotine use in any form.

## Figures and Tables

**Table 1:** Examples of questions asked during the FGIs.

Subject	Leading Questions
Motivation to start smoking/vaping	What made you start smoking/vaping? When did you have your first cigarette? How did you feel then? How many students smoke/vape at your school?
Patterns of using tobacco products	How long do you smoke/vape? What types of cigarettes do you smoke? Why those? How often do you smoke/vape? How much do you smoke/vape? Where do you smoke/vape? When do you smoke/vape? When/where you don’t smoke/vape?
Perception of smoking/vaping and smokers/vapers	How would you describe a smoker/vaper and non-smoker/vaper?
Attitudes toward nicotine addiction	Why do you smoke/vape? How do you feel while smoking/vaping? What do you like/don’t like about smoking/vaping? Does smoking/vaping help you?

**Table 2:** Focus Group Interviews (FGI) distribution.

Gender	Nicotine Users (NU)	Non-Nicotine Users (NNU)
Female	2 FGI Conducted: Lublin 30.10.2019 Warsaw 29.10.2019 total of 16 participants	1 FGI Conducted: Kielce 30.10.2019 total of 8 participants
Male	1 FGI Conducted: Lublin 30.10.2019 total of 8 participants	2 FGI Conducted: Kielce 30.10.2019 Warsaw 29.10.2019 total of 16 participants

## Data Availability

Not applicable.
